# How Microbial Community Composition Regulates Coral Disease Development

**DOI:** 10.1371/journal.pbio.1000345

**Published:** 2010-03-30

**Authors:** Justin Mao-Jones, Kim B. Ritchie, Laura E. Jones, Stephen P. Ellner

**Affiliations:** 1 Department of Ecology and Evolutionary Biology, Cornell University, Ithaca, New York, United States of America; 2 Mote Marine Laboratory, Sarasota, Florida, United States of America; University of York, United Kingdom

## Abstract

Reef coral cover is in rapid decline worldwide, in part due to bleaching (expulsion of photosynthetic symbionts) and outbreaks of infectious disease. One important factor associated with bleaching and in disease transmission is a shift in the composition of the microbial community in the mucus layer surrounding the coral: the resident microbial community—which is critical to the healthy functioning of the coral holobiont—is replaced by pathogenic microbes, often species of *Vibrio.* In this paper we develop computational models for microbial community dynamics in the mucus layer in order to understand how the surface microbial community responds to changes in environmental conditions, and under what circumstances it becomes vulnerable to overgrowth by pathogens. Some of our model's assumptions and parameter values are based on *Vibrio* spp. as a model system for other established and emerging coral pathogens. We find that the pattern of interactions in the surface microbial community facilitates the existence of alternate stable states, one dominated by antibiotic-producing beneficial microbes and the other pathogen-dominated. A shift to pathogen dominance under transient stressful conditions, such as a brief warming spell, may persist long after environmental conditions have returned to normal. This prediction is consistent with experimental findings that antibiotic properties of *Acropora palmata* mucus did not return to normal long after temperatures had fallen. Long-term loss of antibiotic activity eliminates a critical component in coral defense against disease, giving pathogens an extended opportunity to infect and spread within the host, elevating the risk of coral bleaching, disease, and mortality.

## Abstract 2

Modeling reveals how rapid overgrowth by pathogenic microbes in the mucus layer surrounding corals, which often occurs under temporary stressful conditions, can persist long after environmental conditions return to normal.

## Author Summary

An important correlate in bleaching and disease in reef-building corals is a shift in the makeup of the microbial community in the mucus layer surrounding the coral. Resident microbes critical to the healthy functioning of the coral organism are outcompeted by pathogenic microbes, often species of the *Vibrio* bacteria, and usually in the context of environmental disruptions such as ‘heat waves’ during the warm summer months. In this study we introduce mathematical models for microbial community dynamics in the mucus layer to explore how the surface microbial community responds to changes in environmental conditions, under what circumstances it is vulnerable to pathogen overgrowth, and whether it can recover. Consistent with observations that antibiotic properties in coral mucus did not return to healthy, normal levels for many months after temperatures had fallen, we discover that the shift to pathogen dominance under transient stressful conditions may persist long after environmental conditions return to normal.

## Introduction

Reef coral cover has declined dramatically worldwide, due in part to infectious disease outbreaks [1]. This decline was first observed in the wider Caribbean, including the Florida Keys, in the mid- to late 1970s [2]. What permits the spread of infections remains uncertain [1], but many disease outbreaks involve opportunistic infections by endemic microbes following periods of stress [3]–[5]. Many coral disease outbreaks have been preceded by temperature stress and bleaching (loss of photosynthetic algal endosymbionts) [1],[2],[6],[7]. For example, a severe thermal anomaly in the Caribbean in 2005 caused widespread bleaching followed by spread of white plague and yellow blotch diseases, culminating in a 26%–48% loss in coral cover in the U.S. Virgin Islands [6]. This event also demonstrated that disease susceptibility increased in the threatened coral, *Acropora palmata*, after bleaching stress [8]. In addition, a positive correlation has been shown between the frequency of warm thermal anomalies and the occurrence of white syndromes on Australia's Great Barrier Reef [9]. The 1998 El Niño caused widespread bleaching in the western Indian Ocean, followed by 50%–60% mortality in corals [10],[11]. Bleached corals are additionally vulnerable because loss of algae reduces the concentration of oxygen and the resulting radicals that protect the coral animal [12].

Critical to coral disease transmission—or resistance—is the coral's surface mucus layer, which is produced in part by the coral's endosymbionts [13]. The mucus layer hosts a complex microbial community, referred to hereafter as the surface microbial community (SMC). Because the mucus environment is rich in nutrients, microbial population densities there are orders of magnitude higher than in the surrounding water column [13]. It is thought that most established and emerging pathogens are endemic to the ecosystem and typically present at low numbers in the SMC. When stressed, the SMC can switch rapidly from a community associated with healthy corals to one associated with disease [14]–[16]. The mechanisms facilitating this switch, and the underlying population dynamics of microbial species within the coral surface layer, are yet to be elucidated and are the subject of this paper.

Coral mucus provides the nutrient substrate for both beneficial microbes [13],[17]–[21] and pathogenic invaders [13],[22]–[26]. Thus, it is reasonable to expect that altered conditions may shift SMC composition, facilitating pathogen invasion and opportunistic infections. Rapid shifts to pathogen dominance have been observed in the SMC following heat stress [14] and prior to bleaching [5],[27]–[31]. Vega Thurber [14] exposed *Porites compressa* corals in the laboratory to four stressors including temperature stress and observed a shift from “healthy-associated” coral microbiota to a community associated with diseased corals. In field studies during the 2005 summer bleaching event, Ritchie [30] observed that “visitor” bacteria (bacterial groups otherwise not dominant) became the predominant species in mucus collected from apparently healthy *Acropora palmata*. Rosenberg et al. [4] recently summarized evidence supporting a “microbial hypothesis of coral bleaching,” that bleaching is initiated by a shift to pathogen dominance in the SMC brought on by heat stress, rather than primarily by direct effects of heat stress on the coral and its symbionts [32],[33].

In an assay designed to differentiate between “visitor” and “resident” bacteria, it was found that while some visitors produced antibiotic compounds (16% of isolates tested), antibiotic production by coral mucus residents was significantly higher (41% of isolates tested [15]). These results suggest that resident microbes may play an important role in limiting the abundance of pathogenic microbes in the SMC under normal conditions. Consistent with this, densities of beneficial microbes appeared to decrease at times of increased water temperature, when less than 2% of bacteria isolated from the surface of *Acropora palmata* displayed antibiotic activity, significantly less than the 28% of isolates that produced antibiotics in cooler months [15].

A number of qualitative models have been proposed to explain the causes and dynamics of the loss of antibiotic activity and subsequent pathogen dominance in the SMC, focusing on *Vibrio* spp. as a model system for emerging coral pathogens. It is uncertain how stressors facilitate the shift in dominance, but decline of beneficial bacteria is coincident with overgrowth of *Vibrio* spp. and has been observed to precede bleaching and disease outbreak [5],[30],[31],[34]. Ritchie [30] suggested that antimicrobial properties of coral mucus are temperature-sensitive, perhaps due to inactivation of antibiotics with heat or to sensitivity of resident microbes to temperature change. Another hypothesis proposes that *Vibrio* spp., which thrive at elevated temperatures, out-compete beneficial bacteria in these conditions, and a loss of antibiotic activity follows [27]–[30]. These models agree with the experiments of Ducklow and Mitchell [22] in which microbial populations increased significantly when the coral was stressed. This is also true of black band disease, where a diverse assemblage of microorganisms invades and diversity increases with the onset of the disease [35]–[37]. Foster [38] proposed that the competition for space by invasive microbes segregates the beneficial microbes into isolated patches, thereby limiting benefits to the host. Common to all hypotheses is that disease susceptibility is positively correlated with change in SMC composition, loss of antibiotic activity, and an increase in pathogenic microbes.

### Why Take a Modeling Approach?

It appears that antibiotic activity and the competition between beneficial and potentially pathogenic microbes such as *Vibrio* spp. are key to understanding community dynamics within the SMC. In this paper, we develop models for how these interactions affect the outcome of competition within the SMC and either limit or promote overgrowth by pathogenic microbes. We assume that interactions can be simplified to a few key players, each representing some set of microbial organisms or substances within the mucus layer. Some parameter values and model assumptions are based on taking *Vibrios* as a model system for coral pathogens, because less is known about other pathogens of current concern. Yet because many characteristics of the SMC are still uncertain, we explore general properties of the model by varying parameters, rather than attempting to closely simulate any specific coral-pathogen interaction. We thereby identify the parameters and processes that are predicted by the model to have the most significant impacts on SMC dynamics and on the potential for pathogen outbreaks.

We describe first a model that assumes a spatially homogeneous (“well-mixed”) mucus layer. This model does not depict the physical processes of mucus production by the coral or endosymbionts or the loss of mucus by sloughing. Furthermore, gradients in chemical concentrations and in microbial abundances within the mucus layer may have a considerable effect on the qualitative dynamics of the microbial community (as has been observed in other models of interacting microbial populations [39]–[41] and experimentally [42]). We therefore develop a model that includes the spatial gradient in nutrient and microbe concentrations from coral surface to the surrounding seawater, mucus production by the coral, and ablation of mucus into the surrounding seawater. By contrasting this model with the well-mixed model, we examine the role of spatial gradients in SMC dynamics and in defense against pathogen invasion.

## Results

### The Well-Mixed Model

Our well-mixed model and its underlying mechanistic assumptions are presented in detail in the Materials and Methods section. To gain insight into the dynamics of our well-mixed model, we consider a generalized well-mixed model whose long-term properties can be determined by a simple nullcline analysis (nullclines are defined below). Two important aspects of the general model that simplify the analysis are: (1) Microbial populations are measured in units of the growth-limiting substrate provided by the host and its endosymbionts. For example, if we posit that the limiting factor is carbon, then the units for microbial abundance are grams carbon (rather than total biomass, total biovolume, or number of individuals). The model is then nondimensionalized by choosing units such that the total amount of limiting factor in microbes, antibiotics, and the mucus is 1. (2) We assume for now that there is no microbial inoculation from external sources (we later return to the situation in which external inoculation occurs and show that it has no important effects). Because microbes are far less abundant in seawater than in the SMC, inoculation is a small perturbation whose only effect is to prevent complete extinction of either pathogens and beneficials (this is explained in more detail below).

The general well-mixed model is



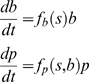
(1)where *p* is the abundance of pathogenic microbes and *b* is the abundance of antibiotic-producing beneficial microbes plus antibiotics, all measured in substrate units as noted above. The amount of free limiting substrate available for microbial uptake and reproduction is then *s* = 1 − *p* − *b*, due to the rescaling such that the total amount of limiting substrate equals 1. Bacterial growth-rate functions ƒ*_p_* and ƒ*_b_* are increasing functions of substrate *s*, and ƒ*_p_* is a strictly decreasing function of *b.* We assume (as is true asymptotically in our specific mechanistic model) that antibiotic concentration is a constant fraction of *b.* We assume growth rates ƒ*_p_* and ƒ*_b_* are both positive when substrate is at its maximum possible value (*s* = 1), because otherwise the populations die out. We also assume that ƒ*_p_* and ƒ*_b_* are both negative when *s* = 0, so neither population can persist in the absence of nutrients.

We can see how model (1) behaves by plotting its nullclines in the (*b*,*p*) plane. The *b* nullcline is the line 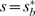 where 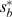 is the solution of 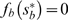, i.e., the line



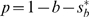
(2)which has constant slope of −1. The *p* nullcline is the curve



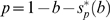
(3)where 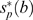 is the solution of 
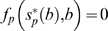
. Because antibiotics are harmful to the pathogens, 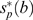 is an increasing function of *b*. Therefore the *p* nullcline has a negative slope that is always below −1.

**Figure 1:**
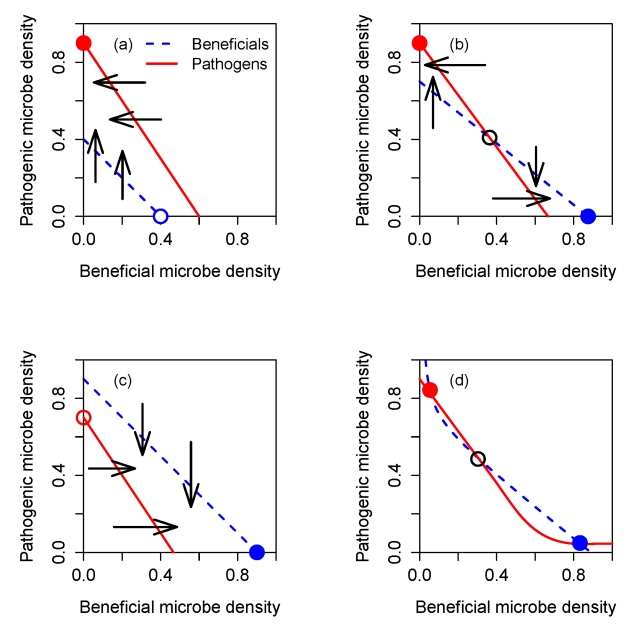
Nullcline analysis of the general well-mixed SMC model. Stable equilibria are shown as filled circles and unstable equilibria as open circles. Arrows indicate the direction of population change on the nullclines. (A) The *P* nullcline (solid red line) lies above the *B* nullcline (blue dashed line), leading to exclusion of beneficials. (B) Because the *P* nullcline is steeper than the *B* nullcline, if the nullclines cross their intersection (open circle) is a saddle and therefore unstable. The stable manifold of the saddle divides the interior of the quadrant into the sets of initial points leading to competitive dominance by one type of microbe and competitive exclusion of the other. (C) The *B* nullcline lies above the *P* nullcline, leading to exclusion of pathogens. (D) Small rates of microbial immigration are permitted, with the qualitative effect of preventing complete extinction when interactions within the mucus layer would otherwise lead to competitive exclusion.

Consequently, there are only three possible qualitative behaviors (Figure 1). If one nullcline lies completely above the other, then all initial conditions with beneficials and pathogenic microbes both present lead to competitive exclusion of the population with the lower nullcline, exactly like the Lotka-Volterra model (Figure 1A, 1C). If the nullclines cross, their intersection is a saddle (locally unstable), so there is competitive exclusion again but with the identity of the winner depending on initial conditions (sometimes called *contingent exclusion*). Both of the single-species equilibria (on the coordinate axes) are then locally stable (Figure 1B).

Without the antibiotic-mediated interactions, we would have pure resource competition for a single limiting substrate. The *p* and *b* nullclines would then be parallel lines, and the pathogen is the superior competitor if the *p* nullcline is above the *b* nullcline (as in Figure 1A). Adding antibiotic effects, if they are sufficiently strong, will make the *p* nullcline decrease more quickly as *b* increases, giving the situation shown in Figure 1B. Control of potentially dominant pathogens through antibiotic activity is thus the “recipe” for contingent exclusion. A numerically dominant pathogen population can prevent regrowth of beneficials by pure resource competition alone, keeping free substrate too scarce for beneficials to increase. A numerically dominant beneficial population can prevent pathogen regrowth by a combination of resource competition and antibiotic production: by maintaining high ambient antibiotic concentration as well as by consuming substrate.

Finally, we can return to the biologically realistic situation in which there is some inoculation of microbes from the surrounding seawater. The empirical observation that microbes are orders of magnitude less abundant in seawater than in mucus implies that external inputs are a small perturbation. Consequently, their only qualitative effect is to prevent complete extinction when interactions within the mucus layer would lead to competitive exclusion. That is, stable equilibria on the axes as replaced by stable equilibria near the axes; unstable equilibria on the axes are eliminated while interior unstable equilibria move to slightly different locations. Figure 1D shows an example of how small rates of immigration change the nullclines and how the equilibria are affected.

### Pathogen Invasion

We now explain how the properties of the well-mixed model can lead to a sudden and persistent “takeover” of the SMC by pathogens following a brief period of conditions stressful to the host and to beneficial microbes. To illustrate the process, we consider thermal stress, which has been implicated most consistently as the environmental driver linked to pathogen outbreaks. External inoculation of microbes is ignored to simplify the presentation, but readers should keep in mind how inoculation would modify the dynamics (as in Figure 1D).

**Figure 2:**
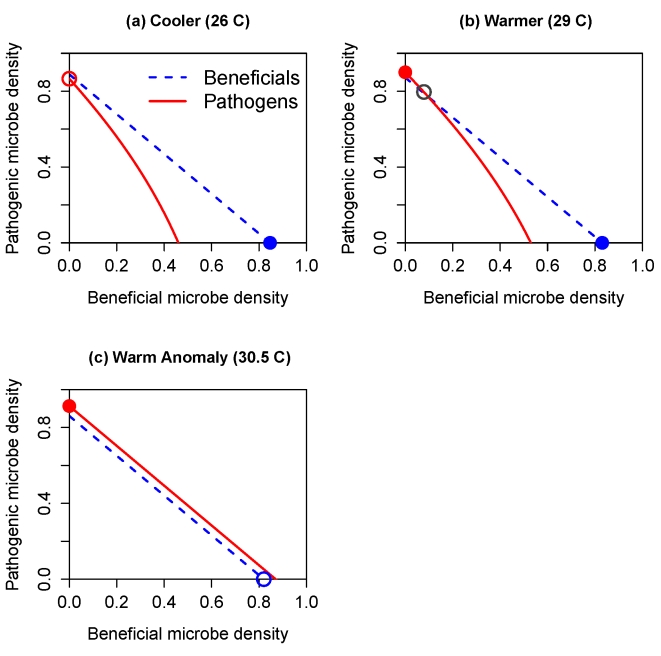
Nullclines (solid red  =  pathogens, dashed blue  =  beneficials) of the well-mixed SMC model (Equation (8)), for the parameter values such that a brief thermal anomaly allows pathogens to become dominant (the slower baseline parameters in Table 1). Panel (A) shows conditions in winter, when the beneficial-dominant equilibrium is stable (solid circle) while the pathogen-dominant equilibrium is unstable (open circle). Panel (B) shows conditions in summer, when the beneficial-dominant and pathogen-dominant equilibria are both stable (solid circles), while the coexistence equilibrium (open circle) is unstable. Panel (C) shows the effect of a small increase in temperature that eliminates antibiotic activity, so that the nullclines of pathogen and beneficial bacteria become parallel. The beneficial-dominated equilibrium (open circle) becomes unstable, so the community converges to the pathogen-dominated equilibrium (solid circle).

For a healthy coral we can assume that during colder months that are unfavorable to the pathogens, the beneficial bacteria are able to outcompete and exclude the pathogens (Figure 2A)—only the pathogen-exclusion equilibrium is stable. During warmer months (Figure 2B) higher temperatures may give the pathogens a higher intrinsic growth rate than the beneficials, but pathogen growth is kept in check by the effect of antibiotics, so the pathogen-exclusion equilibrium remains locally stable. However, a thermal anomaly (transient, unusually high temperature) causes the loss of antibiotic activity (Figure 2C) and eliminates the coexistence equilibrium, so the system jumps to being pathogen-dominated.

**Figure 3:**
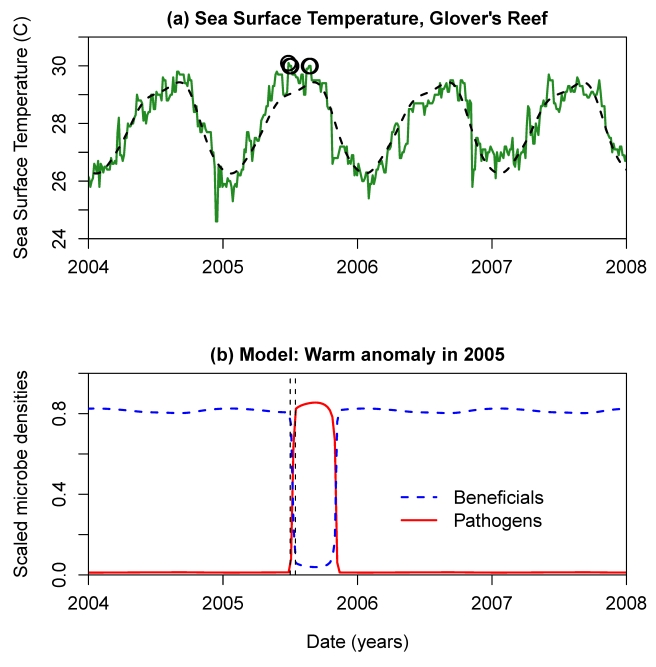
Effects of a brief thermal anomaly on microbial population dynamics in the well-mixed SMC model. (A) NOAA sea surface temperature record for Glover's Reef, Belize (from coralreefwatch.noaa.gov/satellite/data_nrt/timeseries/all_Glovers.txt). The open circles show temperatures considered high enough to elevate the risk of coral bleaching; the dashed curve is the fitted seasonal trend (a periodic smoothing spline) used to simulate the model (B). Simulations of the model using the seasonal temperature trend plotted in panel (A), but with a 2-wk thermal anomaly (indicated by the vertical dashed lines) during which temperature was elevated by 1 degree C, and antibiotic activity by beneficials was eliminated.

Figure 3 illustrates the corresponding microbial population dynamics during a warm anomaly scenario based on sea surface temperatures at Glover's Reef, Belize (Figure 3A). Under normal seasonal variation in temperature, beneficial microbes are dominant year-round (Figure 3B). Regardless of how a simulation is initiated, during winter the beneficials become dominant and they remain dominant through the summer, while pathogens persist at very low levels due to inoculation from the water column. But even a brief thermal anomaly that eliminates antibiotic activity for 14 d (Figure 3B) allows the pathogens to become dominant and remain so for approximately 3 mo, until temperatures drop low enough that the pathogen-dominant equilibrium becomes unstable.

The well-mixed model therefore provides a mechanistic explanation for the empirically observed sudden switches to pathogen dominance following a change in conditions, and moreover the model predicts that such sudden switches will occur even if the change in conditions is gradual. Another key prediction is that the return from pathogen-dominance to beneficials-dominance when conditions improve will also be sudden, but it will occur under different conditions: pathogens may remain dominant even after environmental conditions return to those where beneficials were initially dominant, while beneficials may not recover dominance until the environment becomes considerably more favorable for them. Below, we present experimental results supporting the prediction that beneficial populations may not recover even long after the environmental conditions leading to pathogen takeover have abated.

### The Spatial Model: Qualitative Properties

The fundamental question addressed by the spatial model is whether spatial variability allows for a broader range of qualitative outcomes than the well-mixed spatially homogeneous model. In particular, spatial variability might allow stable coexistence of pathogenic and beneficial microbes, for example if pathogens segregate away from beneficials and so avoid the effects of antibiotics produced by the beneficials. The well-mixed model's prediction of potentially abrupt changes in community composition in response to gradual changes in environmental conditions might then prove to be an artifact of neglecting spatial variability. We therefore expand the model to include spatial variability within the mucus layer along the gradient from host surface to seawater. The model and its underlying assumptions are presented in Materials and Methods, while details of numerical analysis and simulation methods are in the Supporting Text.

**Figure 4:**
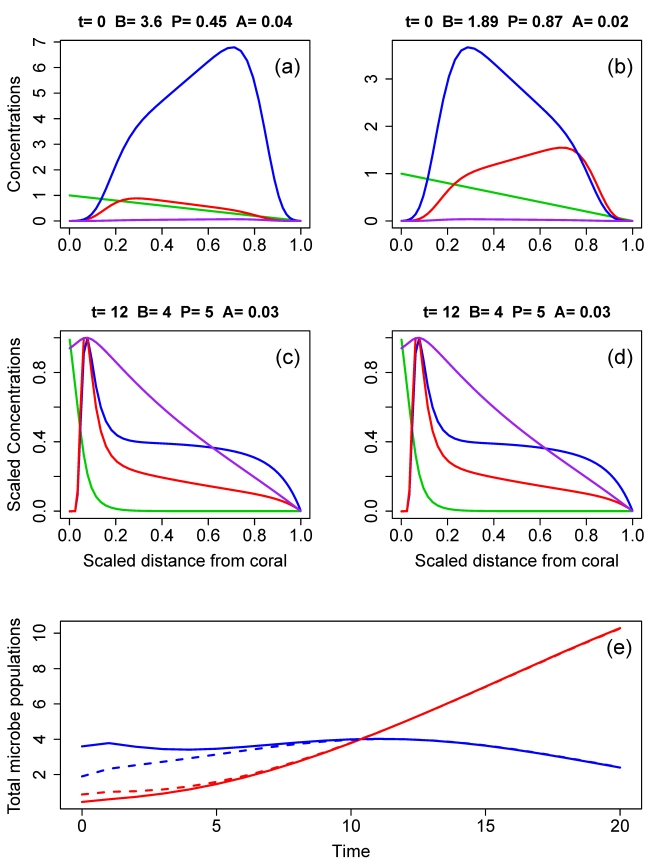
Two runs of the spatial model starting from different initial conditions. Panels (A) and (B) show the initial abundances of all state variables (beneficials:blue, pathogens:red, substrate:green, antibiotic:purple) for the two runs; note that because the model has been rescaled so as to be nondimensional (Text S2), the state variables are unitless. The headings on the panels give the total abundances (P  =  pathogens, B  =  beneficials, A  =  antibiotic). The initial total abundances of beneficials and pathogens for the two runs were chosen so that, although the shapes of the initial distributions differed greatly between the two runs, both give total microbial populations very close to *B*(*t*) = 4, *P*(*t*) = 5 (in the scale of the rescaled spatial model) at time *t* = 12. Panels (C) and (D) show the abundances of all state variables at time *t* = 12 for the two runs, which are nearly identical; in these panels, all state variables have been plotted relative to their maximum value at that time, so that the spatial distributions of all state variables are clearly seen. Panel (E) shows the dynamics of total microbial populations (solid: the run starting from panel (A), dashed: the run starting from panel (B)), illustrating how the two runs converge onto the nearly same point on the intertial manifold and therefore remain nearly identical for all subsequent time.

Numerical study of the spatial model shows that the results from the nullcline analysis of the nonspatial model (Figure 1) continue to hold. Specifically, the spatial model behaves like a two-dimensional system of differential equations, even though it has an infinite-dimensional state space. This occurs because, apart from a brief transient period, the entire spatial distribution of all of the state variables is predictable from the total abundances of beneficial and pathogenic microbes. Figure 4 shows an example. Two model simulation runs were initialized by choosing two different shapes for the spatial distributions of the beneficial and pathogen populations at time *t* = 0 (Figure 4A and 4B), and then finding (using numerical optimization) total population sizes at *t* = 0 such that the total beneficial and pathogen populations at time *t* = 12 would be, for example, 4 and 5, respectively (to within 0.001 or smaller). The outcome (Figure 4C and 4D) is that the two runs are nearly identical in all respects at *t* = 12, not just in their microbial population totals. Beneficials and pathogens are aggregated near the host surface (*x* = 0) where substrate is provided, and where the substrate concentration is sufficient for reproduction to occur. The (lower) microbe abundances further from the host surface are mostly the result of populations being carried along by the mucus. In technical terms, the fact that the two runs have become nearly identical in all respects at *t* = 12 shows that the model has converged quickly onto a two-dimensional *inertial manifold* [43]. On the inertial manifold, the total abundances of the microbial populations are sufficient information to determine the complete state of the system: there is only one way (on the manifold) to have total *B* = 4 and total *P* = 5, and both runs reached that state at *t* = 12. Convergence onto the inertial manifold at time *t* = 12 does not mean that the system has reached equilibrium. As time goes on (Figure 4E) the system state continues to move within the inertial manifold, the pathogens continuing to increase and eventually excluding the beneficials, with both runs following the same path.

**Figure 5:**
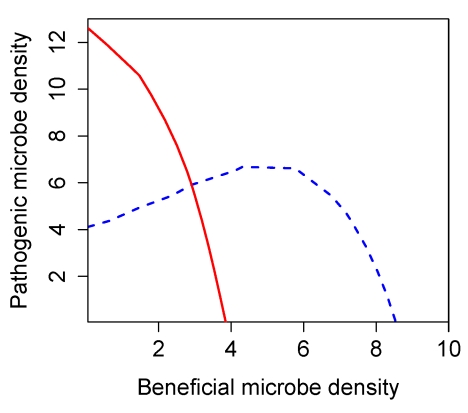
Numerically computed nullclines (beneficials: dashed blue curve, pathogens: solid blue curve) for the spatial model with the slower growth “baseline” parameter values listed in Table 2. The configuration of the nullclines implies stability of both the pathogen-only and the beneficials-only equilibria, with an unstable equilibrium at the intersection of the nullclines.

Convergence of model solutions onto an inertial manifold means that the long-term outcome of the beneficial-pathogen interaction is completely determined by the long-term dynamics on the manifold. For any value (*B*(*t*),*P*(*t*)) of the total microbe populations, there is a unique corresponding system state on the inertial manifold and corresponding instantaneous rates of total population growth *dB/dt* and *dP/dt*. This correspondence defines a two-dimensional dynamical system for the total beneficial and pathogen populations (that is, *dB/dt* and *dP/dt* are both functions of just *B* and *P*), and its behavior can be determined by plotting the nullclines (using the methods described in Text S3). Figure 5 shows nullclines for the slower “baseline” parameters listed in Table 1. These confirm that the spatial model is in the bistable situation of Figure 1(B), indicating that a healthy population of beneficial microbes can keep pathogens from increasing, but beneficials would be at a competitive disadvantage in a community dominated by pathogenic microbes. Given the large uncertainties in our parameter estimates, we cannot regard this property as a prediction about nature. The important feature of Figure 5 is that, as in the well-mixed model, the pathogen nullcline is steeper than the beneficials nullcline, which is the property that precludes robust stable coexistence of beneficials and pathogens with both types abundant (versus low-level persistence of a weaker competitor in the presence of a dominant, due to low-level inoculation from the water column). Consequently, the spatial model preserves the key qualitative prediction of the well-mixed model: if temporary extreme conditions allow the community to become dominated by pathogenic microbes, the pathogen takeover may persist even after conditions return to normal and may not terminate until conditions occur that are highly unfavorable to the pathogens, such as winter temperatures.

**Table 1:** Model parameters and their baseline values (see Text S4) for sensitivity analysis of the spatial model, after rescaling substrate concentration so that the concentration of substrate in fresh mucus supplied by the host is *S*_0_ = 1.

Parameter	Biological Meaning	Units	Default Value	Range
*δ*	Mucus advection rate	mm	0.1	×(0.5–2)
*α*	Fraction of substrate uptake by beneficials that is used to produce antibiotic	Unitless	0.05	×(0.5–2)
*r_B_*	Beneficial maximal growth rate	1/d	0.8, 5	×(0.5–1.5)
*r_P_*	Pathogen maximal growth rate	1/d	1, 6	×(0.5–1.5)
λ	Antibiotic efficacy	1/*C*	5, 44	×(0.5–2)
*k_B_*	Beneficials' half-saturation constant	*C*	0.25	×(0.5–2)
*k_P_*	Pathogens' half-saturation constant	C	0.25	×(0.5–2)
*μ_A_*	Degradation rate of antibiotic	0.5/d	0	×(0.5–2)
*D_S_*	Substrate diffusion coefficient	mm^2^/d	0.1	×(0.5–2)
*D_A_*	Antibiotic diffusion coefficient	mm^2^/d	0.1	×(0.5–2)
*D_B_*	Beneficial microbes diffusion coefficient	mm^2^/d	.01	×(0.5–2)
*D_P_*	Pathogenic microbes rate of diffusion	mm^2^/d	.01	×(0.5–2)
*η_B_*	Beneficial microbes advection coefficient	mm/d	0.05, 0.01	×(0.5–2)
*η_P_*	Pathogenic microbes advection coefficient	mm/d	0.05, 0.01	×(0.5–2)

The two values for parameters *r_B_*, *r_P_*, *η_B_*, *η_P_* are the “slower growth” and “faster growth” baseline parameters for the spatial model discussed in Text S4. The *r* values correspond to the situation in Figure 1B and 2B, where temperatures are warm enough that pathogens have the higher intrinsic growth rate but can be held at low levels by beneficials through antibiotic production and resource competition. *C* in the table denotes units of substrate relative to *S*_0_. The two values of *λ* are for the spatial and non-spatial models, respectively.

### Sensitivity Analysis of the Spatial Model

We performed a local sensitivity analysis to determine the relative impact of each parameter on system dynamics. Due to the high uncertainty of parameter estimates, parameters were varied up to ±50% from their default values (Table 1) using Latin Hypercube sampling (see Appendices D and E for additional information about our sources for parameter values and the methods used to carry out the sensitivity analysis).

We carried out sensitivity analysis under three different scenarios: baseline (the parameter values listed in Table 1), heat stress, and high antibiotic conditions. Baseline parameters correspond to the situation in Figures 1B and 2B, where temperatures are warm enough that pathogens have the higher intrinsic growth rate, but can be held at low levels by beneficials through antibiotic production and resource competition. For the heat stress scenario, beneficials growth rate was reduced, pathogen growth rates increased, and the production of antibiotics was decreased. For the high antibiotic scenario, the antibiotic production rate was increased and the efficacy of the antibiotics against the pathogens increased. We also considered both the “slower growth” and “faster growth” values for the microbe growth rate parameters *r_B_*,*r_p_*. Parameter values for these scenarios are listed in Table 2.

**Table 2:** Default parameter values for the heat stress and high antibiotic scenarios in the sensitivity analysis of the spatial model.

Parameter	Model Scenario			
	Heat Stress Slower	Heat Stress Faster	High Antibiotic Slower	High Antibiotic Faster
*r_B_*	0.6	4.0	—	—
*r_P_*	1.2	7.2	—	—
*α*	0.02	0.02	0.1	0.1
λ	—	—	20	20

A dash (—) indicates no change from the baseline parameter values listed in Table 1. For all parameters not listed here, the default values for these scenarios were the same as those for the baseline scenario listed in Table 1.

**Figure 6:**
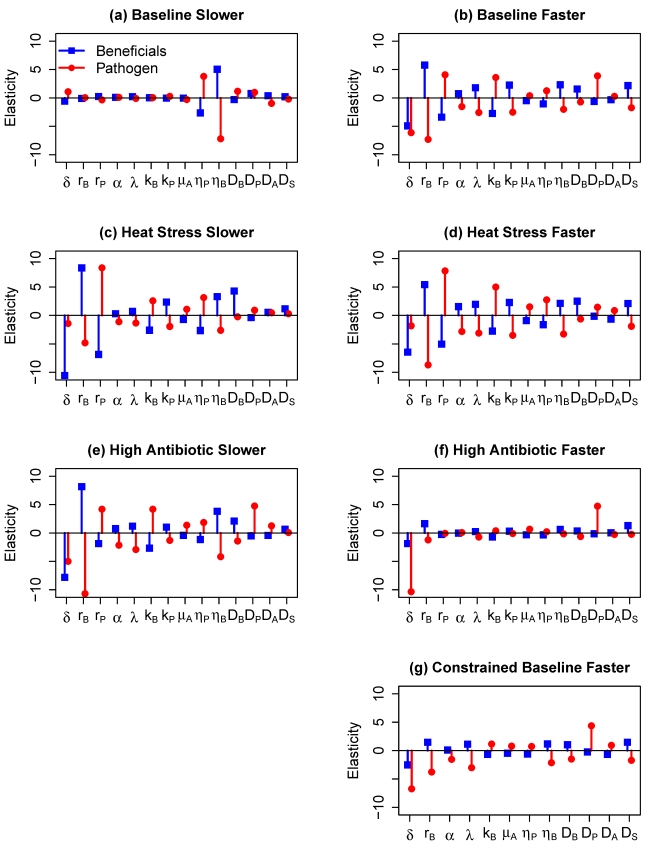
Results of local sensitivity analysis using Latin Hypercube Sampling and multiple linear regression, with parameters allowed to vary by up to ±50% from their default values. See Text S5 for details on methods; parameters and their default values are listed in Table 1. Results are shown for three scenarios (baseline, heat stress conditions, and high antibiotic efficacy) and two sets of values for potential growth rates of the microbe populations (slower and faster). Parameter values for the heat stress and antibiotic scenarios are listed in Table 2. Model responses are the total beneficial (blue) and pathogenic (red) microbe abundances after 30 d (slower parameters) or 20 d (faster parameters). The plotted elasticity values are the regression coefficient for each parameter, in multiple linear regression of ln-transformed response on ln-transformed parameter values. Elasticity values can be interpreted as the average fractional change in the response variable relative to the fractional change in the parameter, so that an elasticity value of 2 means that a ±10% change in the parameter causes a ±20% average change in the response. Note that in panel (G) parameters *k_P_* and *r_P_* are absent, because in the constrained sensitivity analysis these parameters are functions of *k_B_* and *r_B_* (specifically, the ratios *k_P_*/*k_B_* and *r_P_*/*r_B_* are held constant).

Overall, the results of the sensitivity analysis (Figure 6) indicate that the most important parameters are either (1) the advection and diffusion coefficients, which control the balance between active movement towards favorable conditions and mortality through mucus ablation, or (2) the maximum growth rates (*r_B_*,*r_p_*), which are important for the direct competitive interactions between the microbial populations. The importance of advection coefficients (*η_B_* and *η_P_*) reflects our assumption that microbes retain just enough active movement capability to avoid high mortality through mucus ablation, so they are near “tipping point” where a small loss of movement ability has large consequences. Movement parameters were generally less important in the faster growth scenarios (Figure 6B,D,F) where competitive interactions are stronger. Changes in antibiotic production (α) and efficacy (λ) have the most effect on pathogen success in the faster growth baseline (6B) and heat stress scenarios (6D and 6C), because the rate of antibiotic production correlates with the beneficials' population growth rate.

The most surprising outcome is that the parameters controlling the antibiotic production rate (α), degradation rate (μ_A_), and bacteriostatic efficacy (λ) are never among the most significant parameters, even though the beneficials' ability to produce antibiotics is essential for their persistence. This suggests that (if our parameter ranges are realistic) the role of antibiotics in normal conditions is to tip the balance in a competition between near-equals. To explore this idea further, we modified the baseline/faster growth scenario by holding the ratios *r_B_*/*r_p_* and *k_B_*/*k_P_* constant (i.e., for each Latin Hypercube sample parameter vector, we perturbed *r_B_* and *k_B_* and then set the values of *r_P_* and *k_P_* so that the values of *r_B_*/*r_P_* and *k_B_*/*k_P_* were the same in the default and perturbed parameter vectors). As expected, with these constraints (Figure 6G) the importance of growth rate variation (indicated by *r_B_* in the axis label) is greatly reduced relative to that shown in Figure 6B, and the importance of antibiotic-related parameters is greatly increased. The fact that higher overall growth rates are detrimental to the pathogen also reflects the impact of antibiotics, because of the proportionality between beneficials' growth rate and antibiotic production rate in the model.

## Discussion

Previous studies have shown that a sudden shift to pathogen dominance occurs in the SMCs of corals prior to a bleaching event [27]–[30]. It has also been demonstrated that antibiotic activity and antibiotic-producing bacteria in the SMC decline in times of increased water temperature when bleaching is most likely to occur [30]. Disease susceptibility in hard corals is thus positively correlated with a loss of antibiotic activity and an overgrowth of pathogenic bacterial densities in the SMC.

In this article we have developed mathematical models to explore how interactions between resident and invading microbes within the coral SMC affect the health and disease susceptibility of reef-building corals, assuming first a well-mixed mucus layer and then allowing spatial heterogeneity in microbial population densities and nutrient substrate. A surprising but robust finding is the consistency in outcomes between well-mixed and spatially heterogeneous systems. Though spatial heterogeneity might be expected to allow spatial segregation and thus coexistence of pathogenic and beneficial microbe types, in both cases stable coexistence is precluded. Instead, the situation is one of competition and contingent exclusion between two more or less equal competitors, with antibacterial production usually shifting the balance in favor of microbes beneficial to the coral.

### The Well-Mixed Model

Analysis of the well-mixed model shows that under competition for a single limiting substrate, control of pathogen via antibiotic activity is the key to the contingent exclusion of pathogens by the beneficial bacteria. However, under the empirically supported assumption of reduced antibacterial production during heat stress, the model predicts a rapid switch from dominance by beneficial microbes to dominance by pathogens during thermal anomalies. In addition, dominance by beneficials is not restored when temperatures return to the normal conditions under which the beneficials were previously dominant. Instead, conditions must become unusually *unfavorable* to pathogens before a switch back to dominance by beneficial bacterial can occur.

This prediction is consistent with observational findings that antibiotic activity did not return to measurable levels in *Acropora palmata* coral even after recovery and temperature reduction (Table 3). Antibiotic efficacy of coral mucus was assayed by challenging various environmentally relevant sources of microbes against coral mucus. Assays were conducted in April and September of 2005, before (24°C) and during (30°C) one of the highest sea surface temperature increases on record (see Materials and Methods and [30]). Antibiotic activity of mucus sampled from unbleached, apparently healthy areas of *A. palmata* tissue was greatly reduced or eliminated during the September 2005 bleaching event (Table 3). By April 2006 (24°C) most corals on the sampled reef had recovered zooxanthellae, and the *Acropora* were normal in color and not bleached. However, the antibiotic activity of mucus sampled from *A. palmata* in April 2006 remained at levels too low for the assay to detect (Table 3).

**Table 3:** Microbial inhibition by *Acropora palmata* mucus.

	April 2005		September 2005				April 2006		
Microbial Source	Control^a^ (CFU/ml)	Mucus^b^ (CFU/ml)	Antibiotic Strength *θ*	Control (CFU/ml)	Mucus (CFU/ml)	Antibiotic Strength *θ*	Control (CFU/ml)	Mucus (CFU/ml)	Antibiotic Strength *θ*
PDL100	193 (33)	91 (13)	0.75	233 (26)	277 (25)	−0.17	201 (26)	234 (31)	−0.15
Water column	305 (29)	76 (7)	1.4	188 (27)	231 (14)	−0.21	178 (23)	208 (23)	−0.16
Canal water	269 (24)	27 (9)	2.3	328 (28)	274 (20)	0.18	288 (38)	293 (22)	−0.02
African dust	278 (51)	65 (9)	1.45	206 (24)	191 (22)	0.08	271 (21)	273 (28)	−0.01

Results from April and September of 2005 are taken from Table 1 of Ritchie (2006) [30]. Coral mucus was tested for antibiotic activity by plating it directly onto growth media, followed by UV irradiation to inhibit growth of coral-associated microbes. Dilutions of microbial sources containing 100–400 colonies were added to these and to corresponding control plates (also exposed to UV irradiation, but no added coral mucus). CFU/ml values are means (standard deviations in parentheses) of the estimated number of microbial colony forming units (CFUs) for four plates. See Materials and Methods and [30] for additional details. *θ* is a measure of the antibiotic effectiveness of the mucus addition, *θ*  =  ln(Control/Mucus), so a positive value of *θ* means that mucus reduced microbial growth relative to the control, while a value near 0 means that mucus was ineffective at reducing microbial growth.

a Control treatment  =  Growth media + UV treatment.

b Mucus treatment  =  Growth media + mucus + UV treatment.

### The Spatially Heterogeneous Model

Simulations of the spatial model show that the key qualitative result from the analysis of well-mixed model (contingent exclusion) holds in the heterogeneous case as well. However, because of the level of uncertainty in our parameter assumptions and estimations, we performed sensitivity analyses to better quantify the effects of parameter variation under normal warm-season conditions (Figure 6A–B), heat stress conditions (Figure 6C–D), and finally conditions of high antibiotic production (Figure 6E–F).

Under normal conditions, the ability to move towards substrate-rich fresh mucus is critical to the success of a slowly reproducing pathogen, as shown in the relative importance of the advection coefficients *η_P_* and *η_B_*. For a more rapidly growing pathogen under these conditions (Figure 6B), the ability to compete under substrate-limited conditions becomes more important. Under conditions causing heat stress (Figure 6C–D), the pathogen uniformly has the upper hand and its success hinges primarily on its potential population growth rate, *r_P_*. Results for high antibiotic production are similar to baseline conditions, except antibiotic production favors the beneficial bacteria across the entire range of parameters considered. Thus as indicated by sensitivity analysis, the critical parameters overall are those which govern movement (microbial advection and diffusion coefficients and mucus ablation rates) and maximum microbial growth. Finally, a numerical nullcline analysis of the heterogeneous model suggests that it retains the hysteresis observed in the well-mixed model: once temporary extreme heat allows pathogens to overgrow, their dominance will persist until conditions become highly unfavorable for pathogenic persistence.

### Future Questions and Research

Our models have omitted for clarity and simplicity some biological processes important to microbial community dynamics in order to focus on the dynamics between resident and invading bacteria on the coral surface. Our focus on bacterial interactions is motivated by observed correlations between bleaching, coral disease, and a shift in the bacterial community on the SMC of some corals [5],[30],[31],[34]. We thus simplify the interactions in the entire coral holobiont, as interactions between coral zooxanthellae and surface bacteria, marine viruses [42]–[44], and fungi all play a role in coral homeostasis, to see what insight may be gained by focusing on the interactions between resident bacteria and invading pathogens. Though our results address just one important interaction, they nonetheless yield insight into the mechanism behind disease transmission (successful pathogen invasion) in corals.

Our model suggests that higher motility might be very beneficial for microbe populations; if so, temperature-dependent changes in mucus physical properties could be an additional mechanism underlying changes in SMC composition. Indeed, Vega-Thurber discovered that in stressed corals, motility genes associated with *Vibrio* species were dramatically upregulated [14]. Thus, an additional weapon pathogens may have is enhanced motility during pathogenesis, and this might be addressed in a future model.

Future questions include: Why does antibiotic production decline as sea temperatures increase? Are the beneficial bacteria being succeeded by a more temperature-tolerant (and virulent) bacterial type, as suggested by Rosenberg and Ben-Haim [28], who discovered that a *Vibrio* species becomes more virulent and invades at high temperatures; and if so, why? Future models could address the cost of antibiotic production by beneficial bacteria (Does antibiotic production become costly as temperature rises?), incorporate variability in antibiotic conversion efficiency, and allow production of defensive antibiotics by the pathogens themselves, as suggested by Ritchie [30], who showed that visitor microbes, like the *Vibrio* inoculated into the SMC at the mucus-seawater interface, also produce antibiotics.

The present models focus primarily on the loss of defenses within the SMC, without considering the defenses of the host coral itself once an infection has penetrated through the mucus layer. An earlier article focused on cellular immune responses of soft corals to an established fungal infection [44], but did not address how vulnerability to infection is modulated by processes in the SMC. Future models should integrate the process of infection with host immune responses, to address how the onset of pathogenic overgrowth and host cellular response interact to determine the outcome of infection. Fully three-dimensional modeling of the SMC is also important to understand the lateral spread of infections across a colony. But the simplifying assumption of spatially homogeneous conditions (which is typical in theoretical models of spatial population dynamics) is not safe in this case. In our one-dimensional model, the physical processes of mucus creation and loss, and the gradients imposed by the mucus layer's environment, proved to be crucial for all of the model's qualitative predictions. Similarly, realistic modeling of colony modular structure and of spatial variation in multiple limiting factors (where we have assumed single-substrate limitation) may be crucial for modeling the lateral spread or containment of pathogens.

### Conclusions

We have presented models that yield insights into a current crisis in our oceans: the decline of coral cover due to increased vulnerability to disease in a warming climate. Our models show that the physical structure and the nature of the biotic interactions in the SMC facilitate the existence of alternate stable states, one dominated by beneficial microbes and the other dominated by pathogens. This provides a mechanistic explanation for the empirically observed sudden switches to pathogen dominance following heat stress. The models also predict that sudden switches will occur even if the temperature increase is gradual, and that the switch to pathogen dominance will persist long after thermal stress has ceased, so that a short-term heating event may give pathogens an extended opportunity to establish and spread. These predictions are robust consequences of an interaction between beneficial and pathogenic microbes mediated by beneficials' production of antibiotic substances, rather than depending on any fine details of our models. An important practical implication of our findings is that preventing a shift to pathogen dominance (e.g., through amelioration of stressors increasing disease susceptibility such as poor water quality [45],[46] or stimulation of coral immune responses [33]) may be much easier than reversing a shift to pathogen dominance.

## Materials and Methods

### (1) Coral Mucus Inhibition Experiments (Table 3)

Experimental work originally described in Ritchie et al. 2006 [30]. Mucus samples were taken from three apparently healthy *A. palmata* colonies in April of 2005 (mean water temperature of 24°C, sustained at 22–25°C for 2 mo prior to sampling), September of 2005 (mean water temperature of 30°C, sustained at 28–30°C for 2 mo prior to sampling), and April of 2006 (mean water temperature of 25°C, sustained at 23–26°C for 2 mo prior to sampling).

Inhibition assays were carried out by mimicking the coral surface microlayer on growth media by plating 400 microliters of pooled, undiluted coral mucus followed by UV irradiation to kill native mucus-associated microbes. Control media plates were UV irradiated for 10 min to control for UV alteration of media. Environmental sources of potentially pathogenic microbes were diluted and plated onto both mucus treated, and untreated, plates. Potential sources of invasive microbes included *Serratia marscecens* isolate PDL100 that has been implicated in white pox disease of *A. palmata*; Florida Keys canal water collected at sampling intervals; dust from Mali, Africa (collected by V. Garrison); and water column samples collected from the proximity of sampled *A. palmata* colonies during each sampling period. Environmental samples containing viable microbes were serially diluted and plated onto glycerol artificial seawater agar (GASWA) control plates and GASWA + mucus plates. Mucus was tested against all Florida Keys canal water isolates on Luria broth (LB) agar to address resistance to potential water quality contaminants. All other sources were tested on GASWA to address growth inhibition of marine bacteria or microbes implicated as potentially viable in the marine environment. Colony counts were recorded for each experiment and the number of colony forming units (CFUs) per milliliter was estimated.

### (2) A Model for Community Dynamics in a Well-Mixed Mucus Layer

To frame our studies of the SMC, we have developed a simple model for a spatially homogeneous (“well mixed”) mucus layer focusing on the key community members. We explain each state variable and model equation below.

#### Substrate

A nutrient substrate (*S*) is supplied by the host and consists mostly of organic carbon (we use “host” to mean the coral and its endosymbionts together, and the carbon substrate in the mucus is provided primarily by endosymbionts). We assume that substrate is supplied at a constant rate *I_S_* and is consumed only by the modeled populations of beneficial and pathogenic microbes, so the net supply rate as perceived by the populations of interest is constant. Mucus is assumed to slough off at a constant rate *δ* (fraction of mucus lost per unit time). The substrate equation is then:
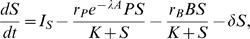
(4)the terms on the right-hand side representing input from the host, uptake by pathogenic and beneficial microbes (discussed below), and mucus loss.

#### Beneficial microbes and antibiotics

We assume the growth of beneficial microbes (*B*) saturates as a function of nutrient concentration, and is described by a Monod equation *S*/(*K* + *S*), where *K* is the half-saturation constant (in units of substrate concentration). We make the simplifying assumption that the substrate content of microbes is released back into the substrate pool immediately upon death, so that net substrate uptake is proportional to the net population growth rate. Maximum per-capita growth rate (as a function of *S*) for the beneficial microbes is denoted 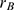. We assume that a constant fraction α of net substrate uptake by beneficials is used to produce antibiotic, and the remainder goes towards population growth. Without loss of generality we scale the microbial populations and antibiotic so that there is a 1∶1 conversion between substrate uptake and population growth or antibiotic production (i.e., we measure microbes and antibiotic in terms of the amount of substrate required to produce them). The equations for beneficial bacteria and antibiotic substances (*A*) are then:
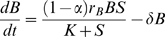
(5)

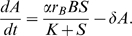
(6)


***Pathogenic microbes*** (*P*) are passively inoculated into the system at rate *I_p_*. Maximum growth rate for the pathogens is *r_P_e* ^−λ*A*^, which is a decreasing function of antibiotic concentration, and growth is described by a Monod equation. We again make the simplifying assumption that the substrate content of these microbes is released back into the substrate pool immediately upon death, so that net substrate uptake is proportional to the net population growth rate:
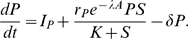
(7)


We now invoke conservation of mass, thus eliminating *S* and *A* as state variables without affecting the model's long-term behavior (see Text S1 for details). Furthermore, scaling the model into dimensionless units gives the rescaled model:
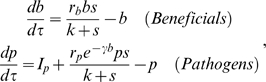
(8)where *s* = 1 − *b* − *p*, and rescaled parameters (such as *r_b_*) are defined in Text S1.

Some biological assumptions of this model should be noted. First, consistent with observations by Ritchie (2009, unpublished data) we assume that the antibiotics produced by beneficial bacteria are bacteriostatic (decreasing the pathogens' ability to reproduce) rather than bacteriocidal (causing pathogen mortality). Second, we assume equal half-saturation constants *K* for pathogens and beneficials. However, the analysis of the general well-mixed model in the text shows that neither of these assumptions affects the qualitative properties of the well-mixed model (so long as, in the alternative assumption that beneficials are bacteriocidal as well as, or instead of, being bacteriostatic, the substrate content of pathogens killed by antibiotics is immediately recycled back into the substrate pool). Third, we do not consider possible effects of oxygen limitation, which might occur at night when the host's photosynthetic endosymbionts are not producing oxygen. Most of the taxa that [30] identified as “resident” are facultatively anaerobic, as are the main potentially pathogenic visitors (*Vibrio* species). For simplicity we thus assume that the microbes of interest can function equally well under anaerobic conditions.

### (3) A Spatial Model for Interactions between Beneficial and Pathogenic Microbes

Spatial structure within the mucus layer is potentially important and inevitably present because of the essential differences between host tissue at the base of the layer that is providing mucus and nutrients and the seawater environment into which mucus and nutrients are lost. We therefore generalize our well-mixed model by allowing spatial variability in the SMC along the gradient from host to water column. In this section we describe the model in some technical detail; readers who wish can omit this section on first reading.

We consider a one-dimensional spatial gradient on the interval 0≤*x*≤1, where the coral surface is at *x* = 0 and the mucus layer meets the water column at *x* = 1. Because mucus is provided at the coral surface and lost by ablation into the water column, we conceptualize the mucus layer as a “conveyor belt” moving from coral to seawater at some constant velocity *δ*. The conveyor belt motion carries along microbes and substrate away from the coral surface (i.e., in the positive *x*-direction), but this is counteracted (in part) by diffusion and by active chemotactic motion of microbes. Because the relative concentrations of substrate, microbes, and antibiotics can vary from one place to another, we cannot reduce the model from four to two state variables. Thus the model tracks the *S*, *P*, *B*, and *A* as functions of space *x* and time *t*. We assume that all particles inside the mucus remain inside the mucus [47] and that no particles diffuse into the water column or through the coral surface. Particles may leave the SMC via mucus sloughing.

At any fixed location *x* within the mucus layer (0<*x*<1), the local interactions are described by the well-mixed model (4–5), but without the supply terms *I_S_* and *I_P_* because these are “active” only at the boundaries. Added to the local interactions are transport terms representing diffusion and advection (directed motion). For the substrate and antibiotics, the transport terms are random Fickian (concentration independent) diffusion and the “conveyer belt” motion at rate 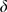, so we have:
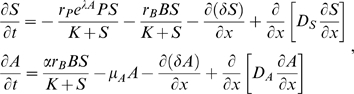
(9)where *D_S_*,*D_A_* are the diffusion coefficients for *S* and *A*, respectively, and *μ_A_* is the antibiotic degradation rate.

The microbes also have diffusion and “conveyor belt” transport terms, and in addition we assume that they are positively attracted to increases in substrate concentration and (for the pathogen) decreases in antibiotic concentration. To represent this mathematically, we posit that the chemotactic velocity component is linearly proportional to the gradient in reproductive rate *W*, where *W* is given in our model by
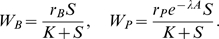
(10)


The microbial dynamics are then
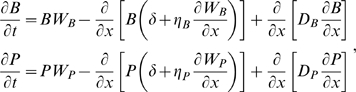
(11)where the chemotaxis coefficients *η_B_*,*η_P_* determine how strongly the microbes respond to gradients in substrate and antibiotic concentration. Flagella are energetically expensive, are often dropped during pathogenesis [48], and are a target of antibody responses, so we assume that microbial motility will be limited, and not much more than the minimum needed to avoid being “swept out to sea” at *x* = 1 through mucus ablation (because substrate is supplied at the coral surface, attraction to substrate automatically favors motion away from *x* = 1).

The differential equations (9) and (11) apply for *x* between 0 and 1, so to complete the model we need to specify what happens at the mucus layer boundaries. Here we give a brief description; see Text S2 for full details and a description of how we numerically solved the spatial model. At the water column boundary *x* = 1, we expect a fairly sharp transition. This can be represented most simply by assuming that anything that reaches the end of the “conveyer belt” falls off it instantly, so the boundary at *x* = 1 is effectively coupled to a void from which nothing returns. We therefore impose the “absorbing” boundary conditions:
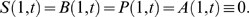
(12)


To allow some immigration from the water column we could set *B*(1,*t*) ≡ *B*_1_,*P*(1,*t*) ≡ *P*_1_ with *B*_1_,*P*_1_<<1. For simplicity we use (12) but recognize that immigration would prevent complete extinction of either beneficial or pathogenic bacteria, as discussed in the main text.

Substrate is supplied at the coral surface, which means in our “conveyor belt” model that new mucus has a high substrate concentration determined by the host. The boundary condition for substrate at *x* = 0 is therefore *S*(0,*t*) ≡ *S*_0_>0. Antibiotic is neither supplied nor absorbed at the coral surface, so the appropriate boundary condition is that there be zero flux across the boundary. The same is true for the microbial populations, but a simple no-flux condition would lead to microbes piling up at the coral surface to get the most possible substrate. This is not observed, perhaps because there is increased viscosity in newly released mucus that would inhibit mobility and keep the microbes from reaching the coral surface. Schneider and Doetsch [49] observed the effect of viscosity on motility under experimental conditions, finding that motility decreased at high and low viscosities and was maximized at intermediate viscosity. Therefore, following [44] we made the boundary at *x* = 0 inaccessible to the microbes by having the diffusion and advection coefficients decrease smoothly to zero near the coral surface.

## Supporting Information

Text S1 **Here, we simplify and rescale the well-mixed model.**
(0.04 MB PDF)

Text S2 **Here, we show how the spatial model can be rescaled into nondimensional form, give additional technical details on the boundary conditions and how they were imposed numerically, and describe our methods for numerical solution of the spatial model [44],[50]–[52].**
(0.03 MB PDF)

Text S3 **Here, we describe how nullclines may be computed numerically for the rescaled spatial model.**
(0.03 MB PDF)

Text S4 **Here, we give and explain the parameter baseline values used in the Sensitivity Analysis [34],[48],[ 53]–[62].**
(0.06 MB PDF)

Text S5 **Here, we give additional methodological details for our sensitivity analysis of the spatial model and a more extensive discussion of the results [63],[64].**
(0.02 MB PDF)

## Abbreviations

CFUs: colony forming units
GASWA: glycerol artificial seawater agar
LB: Luria broth
LHS: Latin Hypercube Sampling
SMC: surface microbial community
