# Supplementary material for: How Microbial Community Composition Regulates Coral Disease Development
Source: PLoS Biol. 2010 Mar 30;8(3):e1000345. doi: 10.1371/journal.pbio.1000345 (PMC2846858; doi:10.1371/journal.pbio.1000345)
Supplement: Text S1 — Here, we simplify and rescale the well-mixed model. (0.04 MB PDF) [file pbio.1000345.s001.pdf]

Appendix A: Simplifying and rescaling the well-mixed model

The total amount of substrate $\Sigma \equiv S + P + B + A$ obeys the differential equation $d\Sigma / dt = I_S + I_P - \delta\Sigma$, and so Σ converges to $\Sigma^* = (I_S + I_P)/\delta$. For studying the long-term dynamics of the model we can therefore assume that Σ has converged to the limiting value, so that $S \equiv \Sigma^* - (P + B + A)$. Also, *A* and *B* are produced in the constant ratio $\alpha : (1-\alpha)$ and have the same proportional loss rate *δ*, so the ratio of *A:B* converges to $c = \alpha /(1-\alpha)$. This allows us to write *A*= *cB*, leaving only *P* and *B* as state variables. The model is then

$$\begin{aligned} \frac{dB}{dt} &= \frac{(1-\alpha) r_B BS}{K+S} - \delta B \\ \frac{dP}{dt} &= I_P + \frac{r_P e^{-\lambda c B} PS}{K+S} - \delta P \end{aligned} \tag{1}$$

with $S = \Sigma^* - (P + (1+c)B), c = \alpha /(1-\alpha).$

To nondimensionalize the model, we rescale the state variables and time as follows:

$$x = (1+c)B / \Sigma^*, y = P / \Sigma^*, \tau = \delta t \,. \tag{2}$$

Note that in this rescaling, *x* represents the total amount of substrate (relative to Σ^*) in beneficials and antibiotic. By standard calculations, these rescalings convert (1) into equation (8) of the main text with scaled parameters

$$r_x = (1-\alpha) r_B / \delta, r_y = r_P / \delta, \gamma = \lambda \alpha \Sigma^*, k = K / \Sigma^*, I_y = I_P /(I_S + I_P) \,. \tag{3}$$
