# Supplementary material for: How Microbial Community Composition Regulates Coral Disease Development
Source: PLoS Biol. 2010 Mar 30;8(3):e1000345. doi: 10.1371/journal.pbio.1000345 (PMC2846858; doi:10.1371/journal.pbio.1000345)
Supplement: Text S2 — Here, we show how the spatial model can be rescaled into nondimensional form, give additional technical details on the boundary conditions and how they were imposed numerically, and describe our methods for numerical solution of the spatial model [44],[50]–[52]. (0.03 MB PDF) [file pbio.1000345.s002.pdf]

Text S2. Technical details of the spatial model

Here we show how the spatial model can be rescaled into nondimensional form, give additional technical details on the boundary conditions and how they were imposed numerically, and describe our methods for numerical solution of the spatial model.

Rescaling. The equations to be non-dimensionalized are

$$\begin{aligned}
\frac{dS}{dt} &= -W_B B + W_P P - \delta \frac{\partial S}{\partial x} + D_S \frac{\partial^2 S}{\partial x^2} \\
\frac{dB}{dt} &= (1-\alpha) W_B B - \frac{\partial}{\partial x}\left[B\left(\delta + \eta_B \frac{\partial W_B(S)}{\partial x} \right)\right] + \frac{\partial}{\partial x}\left[D_B \frac{\partial B}{\partial x}\right] \\
\frac{dP}{dt} &= W_P P - \frac{\partial}{\partial x}\left[P\left(\delta + \eta_P \frac{\partial W_P(S)}{\partial x} \right)\right] + \frac{\partial}{\partial x}\left[D_P \frac{\partial P}{\partial x}\right] \\
\frac{dA}{dt} &= \alpha W_B B - \mu_A A - \delta \frac{\partial A}{\partial x} + D_A \frac{\partial^2 A}{\partial x^2}
\end{aligned} \qquad (1)$$

where $$W_B = \frac{r_B S}{k_B + S}, \quad W_P = \frac{r_P e^{-\lambda A} S}{k_P + S}.$$

We measure time in days, distance *x* in mm, and state variables are all measured in units of substrate (e.g., moles Carbon). The units of parameters are given in Table 2. For simplicity, the spatial dependence of advection and diffusion coefficients used in numerical solutions is not shown explicitly in equations (1).

It is convenient to choose substrate units so that the concentration of substrate in fresh mucus supplied by the host is $S_0 = 1$, because this stabilizes the one non-zero boundary condition (i.e, $S(0,t) \equiv S_0$). A typical value of mucus layer thickness is *L*=1mm so there is no gain from rescaling the spatial variable *x*, and likewise for numerical studies there is no gain from rescaling time. We therefore proceed much as in the nonspatial model, and define rescaled state variables

$$\hat{S} = S / S_0, \hat{B} = B / S_0, \hat{P} = P / S_0, \hat{A} = A / S_0$$

(the scaling of *B* is different here than in the well-mixed model, because we cannot tacitly absorb *A* into *B* when both quantities are varying across space). By standard calculations, the resulting rescaled model is then identical in form to equation (1), except that the values of the parameters k_B and k_P are both divided by S_0.

Boundary Conditions. At the right-hand boundary, the sharp transition between mucus layer and the surrounding water column is represented by absorbing boundary conditions $S(1,t) = B(1,t) = P(1,t) = A(1,t) \equiv 0$. In numerical experiments these gaves very similar results to less extreme boundary conditions (e.g., allowing some two-way diffusion between mucus layer and seawater), so we used the absorbing conditions for simplicity and to avoid introducing additional parameters specifying the boundary conditions.

The left-hand boundary conditions are constant for substrate, $S(0,t) \equiv S_0 > 0$ to represent substrate supply by the host and its symbionts, and zero-flux for the other state variables. For the antibiotic the zero-flux condition is that $-\delta A + D_A \frac{\partial A}{\partial x} \equiv 0$ at *x*=0. In numerical solutions this condition was imposed by finite difference, as detailed below (equation (3)).

Boundary conditions for the microbe populations at *x*=0 are more complicated. Our chemotaxis assumptions favor microbes converging onto the coral surface to maximize nutrient uptake. To avoid this behavior, which is not actually observed, following Ellner et al. (2007) we made the boundary at *x*=0 inaccessible to the microbes

by having the diffusion and advection coefficients decrease smoothly to zero as the coral surface is approached:

$$D_B(x) = D_B^* x^2 / (x_0^2 + x^2), \quad \eta_B(x) = \eta_B^* x^2 / (x_0^2 + x^2) \qquad (2)$$

and similarly for the pathogens, where $0 < x_0 << 1$. The resulting boundary conditions are $B(0,t) = P(0,t) \equiv 0$. Numerical experiments show that the variable coefficient approach, equation (2), gives results very similar to implementing a no-flux boundary conditions by finite difference, but is more stable against numerical blowups that can occur when all microbes concentrate near the coral surface.

Numerical methods. Model solutions were obtained by methods very similar to Ellner et al. (2007, Appendix B). Spatial derivatives of state variables were calculated by Chebyshev interpolation [50] with grid points running from 0 to 1, and we used the "method of lines" to solve the model at interior grid points (i.e. all but *x*=0 and *x*=1). Method of lines produces a system of ordinary differential equations representing state variable values at the interior grid points. The odesolve package [51] in R [52] was used to numerically solve these differential equations.

Values at the boundary grid points are constants specified by the boundary conditions, except for the value of *A* at *x*=0. For this we used a finite difference approximation to the no-flux boundary condition:

$$-\delta(A_0 + A_1)/2 + D_A \frac{A_1 - A_0}{x_1 - x_0} = 0 \qquad (3)$$

(indices 0 and 1 indicate the left-most grid point and its neighbor to the right), and solved equation (3) for A_0 as a function of A_1. Although more complex schemes could be used

that achieve higher-order theoretical accuracy, we have found that finite difference is much more robust against grid-scale numerical artifacts in the interior that affect higher-order methods such as spectral estimates [44].
