# Supplementary material for: How Microbial Community Composition Regulates Coral Disease Development
Source: PLoS Biol. 2010 Mar 30;8(3):e1000345. doi: 10.1371/journal.pbio.1000345 (PMC2846858; doi:10.1371/journal.pbio.1000345)
Supplement: Text S3 — Here, we describe how nullclines may be computed numerically for the rescaled spatial model. (0.03 MB PDF) [file pbio.1000345.s003.pdf]

Text S3. Computing Nullclines of the Spatial Model

Let **B** and **P** denote the total beneficial and microbe densities in the spatial model, i.e. the integrals of $B(x,t)$ and $P(x,t)$ from x=0 to 1, respectively. To find the nullclines, we first computed values of *d***B***/dt* and *d***P***/dt* for the rescaled spatial model, at a grid of values for **B** and **P**, and then used the contour() function in R [52] to approximate the curves in the (**B**, **P**) plane on which *d***B***/dt*=0 and on which *d***P***/dt*=0 .

The derivatives *d***B***/dt* and *d***P***/dt* need to be computed in the asymptotic spatial distribution (such as the bottom row of Figure 4) determined by the specified values of **B** and **P**. To compute the asymptotic spatial distribution for given **B** and **P**, we modified the spatial model so that the spatial processes in the microbe populations continued to operate, but the total population sizes did not change. Specifically, consider a modified spatial model in which terms have been added to the equations for *B* and *P* that are proportional to the current population at each spatial location, giving

$$
\begin{aligned}
\frac{d\tilde{B}}{dt} &= (1-\alpha)W_B\tilde{B} - \frac{\partial}{\partial x}\left[\tilde{B}\left(\delta+\eta_B\frac{\partial W_B(\tilde{S})}{\partial x}\right)\right] + \frac{\partial}{\partial x}\left[D_B\frac{\partial \tilde{B}}{\partial x}\right] + \gamma_B\tilde{B} \\
\frac{d\tilde{P}}{dt} &= W_P\tilde{P} - \frac{\partial}{\partial x}\left[\tilde{P}\left(\delta+\eta_P\frac{\partial W_P(\tilde{S})}{\partial x}\right)\right] + \frac{\partial}{\partial x}\left[D_P\frac{\partial \tilde{P}}{\partial x}\right] + \gamma_P\tilde{P}
\end{aligned}
\qquad (1)
$$

(the tilde's in equation (1) indicate that these are state variables in the modified model). Because the new terms are proportional to current population size, their immediate effect (over a short time-step in numerically integrating the model forward in time) is to change the total population sizes but not the shape of the spatial distribution.

We now want to choose values of the γ's in equation (1) so that the total population size remains constant even though all other aspects of the model, including

changes in spatial pattern, are continuing to operate "as usual". Integrating equations (1) over (0,1) and using the boundary conditions, we have

$$\begin{aligned} \frac{d\tilde{\mathbf{B}}}{dt} &= (1-\alpha)\int_0^1 W_B \tilde{B}(x,t)dx + D_B \frac{\partial \tilde{B}(1,t)}{\partial x} + \gamma_B \tilde{\mathbf{B}} \\ \frac{d\tilde{\mathbf{P}}}{dt} &= \int_0^1 W_P \tilde{B}(x,t)dx + D_P \frac{\partial \tilde{P}(1,t)}{\partial x} + \gamma_P \tilde{\mathbf{P}} \end{aligned} \tag{2}$$

so total population size is held constant by setting the γ's at each time-step of the numerical integration of the modified spatial model such that the right-hand sides of (2) are zero. For this calculation the integrals on the right-hand side of equation (2) were computed by Clenshaw-Curtis integration [50] and the spatial derivatives at x=1 were computed using the Chebyshev differentiation matrix, in both cases using the Chebyshev spatial grid being used for numerical solution of equation (1) by method of lines (as described in the main text).

Given **B** and **P** values, the modified model using (1) for the microbe populations was initialized with initial distributions satisfying the boundary conditions such that the total microbe populations have the specified values. This model was integrated from t=0 to t=12, which was sufficient time for the spatial distributions to converge to a static form, while the total microbe populations were held constant. The original spatial model was then integrated for 0.1 time units, starting from the final state of the modified model. The changes in total microbe density over the 0.1 time units, multiplied by 10, were used as the estimates of $d\mathbf{B}/dt$ and $d\mathbf{P}/dt$ for the original spatial model.
