# Supplementary material for: How Microbial Community Composition Regulates Coral Disease Development
Source: PLoS Biol. 2010 Mar 30;8(3):e1000345. doi: 10.1371/journal.pbio.1000345 (PMC2846858; doi:10.1371/journal.pbio.1000345)
Supplement: Text S4 — Here, we give and explain the parameter baseline values used in the Sensitivity Analysis [34],[48],[ 53]–[62]. (0.06 MB PDF) [file pbio.1000345.s004.pdf]

Text S4: Parameter Baseline Values for Sensitivity Analysis

Thickness of mucus layer. The outer mucus layer can vary in thickness from a few tenths of a millimeter to several millimeters [53]. We therefore assume that mucus layer thickness is about 1 mm.

Intrinsic Rates of Microbe Population Growth. Under ideal conditions in the lab, *in vitro* bacterial growth rates can be extremely high, with doubling times well under one hour. For example, Sharon and Rosenberg [53] observed doubling times of ~30 minutes on diluted coral mucus at 30°C, by bacterial strains identified as *Vibrio* species based on 16S rRNA. This corresponds to an intrinsic growth rate of $r = \log(2^{48}) \approx 33/d$ at 30°C. Applying a Q10 of 2.4 (see below), this corresponds $r \approx 21/d$ at 25°C. These values imply growth by over 9 orders of magnitude within a day. Growth rates on living corals *in situ* are likely to be much lower due to competition with other species and local resource depletion, but to our knowledge there are no empirical estimates available.

We therefore considered two different sets of baseline values for microbial potential growth rates: $r_B = 0.8/d, r_P = 1/d$ ("slower") and $r_B = 5/d, r_P = 6/d$ ("faster"), at typical summer temperatures that are non-stressful to the host (≈29°C or slightly cooler). Both of these allow rapid changes in microbial populations – roughly thousand-fold growth within a week at the slower parameters, and more than million-fold growth within 3 days at the faster parameters. Both parameter sets represent our biological premises about the interaction between beneficials and pathogens: (i) pathogens have the higher intrinsic growth rate in warmer months but are kept in check by the antibiotics produced by the beneficials; (ii) effects of seasonal temperature variations on microbial growth rates (see below) give the beneficials the advantage during cooler months even in

the absence of antibiotics. Without property (i), pathogens would never be able to grow at the expense of beneficials. Without property (ii), hosts could never recover from a pathogen outbreak.

Antibiotic efficacy. Antibiotic activity in coral surface mucus caused a roughly 10-fold reduction in pathogen growth rate at temperatures near 25°C (K. Ritchie, *unpublished data*). In our scaled well-mixed model, beneficials are at a density near 1 (in the model's non-dimensional units) during cooler months, so we need $e^{\lambda c} \approx 10$, which is $\lambda c \approx 2.3$, where $c = \alpha/(1-\alpha)$. In the scaled spatial model, beneficial densities during cooler months are roughly 10 (in the model's non-dimensional units) in the regions near the coral surface where substrate availability is high and microbe population growth occurs, so for the spatial model we need λ smaller by a factor of 10.

Temperature Dependence of Microbe Growth Rates. Kirschner et al. [54] estimated that *Vibrio cholerae* population growth rate was proportional to $e^{0.088T}$ where T is Celsius temperature; the corresponding Q10 value is exp(0.88) ≈ 2.4. Vital et al. [55] found a ratio of 2.1 between *Vibrio cholerae* population growth rates at 30°C versus 20°C, i.e. Q10=2.1. In our simulations that incorporate temperature effects we assumed Q10=2.4 for the pathogenic microbes. In contrast to the evidence that vibrios grow faster at 30°C than at 25°C [54,55,56,57] , laboratory experiments on three beneficial bacteria isolated from *Acropora palmata* (Beck Frydenborg and K. Ritchie, *unpublished data*) found that their growth rates at 30°C were lower (2 isolates) or no higher than (1 isolate) the growth rate at 25°C . It is also known that some *Vibrio* species produce a

photosynthesis inhibitor at elevated temperatures [34]. We do not model this effect explicitly, but assume that elevated temperatures can decrease the growth rate of beneficial microbes.

Microbial diffusion coefficients. Diffusion coefficients have been estimated for *E. coli* in several different media but not (to our knowledge) for other bacteria, so we use those as a rough guide. Berg and Turner [58] estimated three-dimensional diffusion coefficient $D = 5 \times 10^{-6}$ cm^2/sec for wild-type *E.coli* in water, very close to their theoretically predicted value of 4×10^{-6} cm^2/sec. We need to divide this value by 3 to get the diffusion coefficient for the component of motion in one spatial dimension, and multiply by $(60\times60\times24)$ to convert to our model's time units (days). The result is 0.144 cm^2/day. The corresponding diffusion distance (root mean square displacement) over a one day time interval is about 5mm, more than the typical thickness of the mucus layer.

So as with population growth rates, these *in vitro* estimates cannot be applied directly to microbes *in vivo* in coral mucus. There are at least two differences that need to be considered. First, mucus is more viscous than water. Second, wild-type *E. coli* are actively propelling themselves with flagella, and we expect this to be much less important for bacteria in the mucus layer, which may lose their flagella because of their energetic cost [59].

A more relevant estimate may be Berg's estimate for dead or paralyzed *E. coli* in water at room temperature: $D = 2 \times 10^{-9}$ cm^2/sec [60]. This translates to 6×10^{-5} cm^2/day in a single spatial dimension, a root mean square displacement in 1 day of 0.1mm. If we

assume a mucus layer thickness of 1mm, then on the model's length scale (i.e. L=1 being 1mm), this estimate becomes D=0.006.

The higher viscosity of mucus cuts both ways: it would slow down the diffusion of a completely passive microbe, but speed up active propulsion because the thrust generated by a flagellar rotor is proportional to the viscosity of the medium [60]. Assuming that microbes in the mucus layer should be viewed as minimally active swimmers, we have used D=0.01 as a default value for the microbes. This is small enough that even substantial changes should not have much of an impact, because it is in a range where effects of microbial diffusion are dominated by other transport processes: advection up nutrient gradients, and the "conveyor belt" motion of the mucus medium.

Diffusion coefficients for substrate and antibiotic

These diffusion coefficients are estimates due to a lack of information on coral mucus viscosity. A variety of organic compounds, such as sugars and amino acids, have diffusion coefficients in water at 25°C on the order of 10^{-5} cm^2/sec, which converts to roughly 30 mm^2/d (CRC Handbook of Chemistry and Physics, 2007-08 edition at www.hbcpnetbase.com). The viscosity of water is approximately 1 centipoise, while that of human gut mucus at low shear (probably typical of coral mucus) is higher by a factor of about 3000 [61]. The diffusion coefficient is inversely proportional to viscosity, so we have the estimate D=0.01 mm^2/day. Dog gastric mucus has about 50 times the viscosity of water [62]. Like coral mucus it is a mix of carbohydrates and proteins, so this may be a reasonable viscosity estimate for coral mucus, giving the estimate D= 0.6 mm^2/day.

Given this wide range we have used D=0.1 as our default value for both substrate and antibiotics.

Advection coefficients for microbes. Microbes in the mucus layer would be expected to expend the minimum effort needed to keep themselves in preferable regions of the mucus layer, close to the nutrient source (the coral host) rather than the relatively hostile seawater environment. In some host-microbe interactions, once bacteria have colonized the host from the water column they no longer produce flagella [63], presumably to avoid the metabolic costs of synthesizing flagella and flagellar motion (in addition, flagella can be used as antigenic targets by the immune system, but this may not be relevant with an invertebrate host). We therefore set the advection coefficients to values just high enough so that microbe populations at baseline values of the other parameter established a clear gradient in density concentrated near the coral host, but not so large that microbes were able to concentrate exclusively in a narrow band very near the host.

Substrate limitation of microbe growth. Values of the half-saturation coefficients were chosen so that fresh mucus (substrate level S=1 in our scaled models) would put the microbes near saturation, but 90% substrate depletion would have a substantial effect. Given the sharp substrate gradients that are established in the spatial model, going quickly from S=1 to S near zero within a small distance from the coral host, the value of the half-saturation constant should not be critically important within a broad range.
