# Supplementary material for: How Microbial Community Composition Regulates Coral Disease Development
Source: PLoS Biol. 2010 Mar 30;8(3):e1000345. doi: 10.1371/journal.pbio.1000345 (PMC2846858; doi:10.1371/journal.pbio.1000345)
Supplement: Text S5 — Here, we give additional methodological details for our sensitivity analysis of the spatial model and a more extensive discussion of the results [63],[64]. (0.02 MB PDF) [file pbio.1000345.s005.pdf]

Text S5: Sensitivity Analysis Methods and Discussion

Here we give additional methodological details for our sensitivity analysis of the spatial model and more extensive discussion of the results.

Methods: For each scenario, 500 perturbed parameter sets were generated by Latin Hypercube Sampling [64,65]. For each of these we ran the model to compute the total beneficial and pathogenic microbial populations after 30 days (slower growth) or 20 days (faster growth). The relative importance of each parameter was measured by using multiple linear regression to compute the elasticity of the final microbial populations with respect to each parameter. Elasticity is the proportional change in the response relative to the proportional change in the parameter, averaged over the parameter range being considered. So an elasticity value of 2 means that a 5% change in the parameter causes a 10% change in the response variable (total beneficials or pathogens). Elasticity values were estimated by multiple linear regression of log-transformed final microbial abundances on log-transformed parameters, and the slope coefficient for each parameter is the estimated elasticity.

Initial conditions: Different initial conditions were used for each scenario. Baseline scenario initial conditions were chosen to test, for a healthy coral in normal warm-month circumstances, which parameters affected the effectiveness of defense against pathogen invasion and in particular the potential for competitive exclusion of pathogens. The initial conditions therefore had significant concentrations of both beneficials and pathogens to allow opportunity for competitive exclusion by either. To test which parameters affected the coral's degree of vulnerability to pathogen invasion under heat stress, heat stress scenario initial conditions set pathogen initial concentrations

just above zero. High antibiotic scenario initial conditions set pathogens significantly more abundant than beneficials to test the efficacy of the antibiotics as a mechanism for invasion by beneficials.

Results: Baseline Scenario: In the baseline/slower growth scenario (Figure 6a) the most significant parameters are the chemotaxis coefficients η_B, η_P, which regulate the rate at which beneficials and pathogens spread to higher concentrations of nutrients. Success of either the beneficials or pathogens is determined by their ability to overcome the potential mortality due to mucus ablation, and all other parameters are insignificant. With faster microbial growth (Figure 6b), chemotaxis is much less important than parameters affecting the competitive interactions between pathogenic and beneficial microbes. The directions of parameter effects are all as one would expect, for example a higher value of r_B increases the beneficials and decreases the pathogens, while a higher value of δ is detrimental to both. The efficacy of antibiotics (determined by α, λ and μ_A) is masked by the dramatic changes in antibiotic production related to changes in the beneficials growth rate.

Results: Heat Stress Scenario: In the heat stress/slower growth scenario (Fig. 6c) pathogen success correlates most strongly with pathogen maximum growth rate. Beneficials maximum growth rate and half saturation constant, and the chemotaxis parameters are of secondary importance, and all other parameters are insignificant for pathogen success. Beneficials success correlates most with its advection and potential growth rates and with the mucus ablation rate. Because antibiotic production and efficacy are greatly reduced in this scenario, the corresponding parameters (α, λ, μ_A) are unimportant relative to parameters affecting the resource competition between beneficials

and pathogens. Results for the heat stress/faster growth scenario (Fig. 6d) are very similar, except that higher beneficials' growth rate implies higher antibiotic production, so the importance of α and λ is increased. Pathogen success correlates most with growth rates and beneficials' half saturation constant, and beneficials success correlates most with advection and growth rates, with all other parameters being less important or insignificant.

Results: High Antibiotic Scenario: Results for the high antibiotic/slower growth scenario (Fig. 6e) are very similar to the baseline/faster growth scenario. Pathogen success is highly and negatively correlated with the beneficials growth rate. Also significant are advection, pathogen growth rate, beneficials half saturation constant, beneficials chemotaxis, and pathogen diffusion. Beneficials success is highly correlated with advection and beneficials growth rate. In the high antibiotic/faster growth scenario (Fig 6f) the combination of high growth rate, high antibiotic production and high antibiotic efficacy gives the beneficials the advantage across the entire range of parameters considered for the sensitivity analysis. Conditions for the pathogens are uniformly suboptimal (either due to low resource levels or high antibiotic concentrations) resulting in an inability to escape through chemotaxis. The only factor affecting their success is the balance between mortality through mucus ablation and diffusion away from the ablation boundary.
